# Development of a Gold Nanoparticle-Based ELISA for Detection of PCV2

**DOI:** 10.3390/pathogens13020108

**Published:** 2024-01-26

**Authors:** Caroline Rodrigues Basso, Taís Fukuta Cruz, Larissa Baldo Vieira, Valber de Albuquerque Pedrosa, Fábio Sossai Possebon, João Pessoa Araujo Junior

**Affiliations:** 1 Biotechnology Institute, São Paulo State University, Botucatu 18607-440, SP, Brazil; tfcruz@yahoo.com.br (T.F.C.); fabio.possebon@unesp.br (F.S.P.);; 2 Chemical and Biological Sciences Department, Bioscience Institute, São Paulo State University, Botucatu 18618-000, SP, Brazil; valber.pedrosa@unesp.br

**Keywords:** *Circovirus porcine2*, ELISA, gold nanoparticles, PCV2

## Abstract

In this new methodology, plasmonic ELISA (pELISA) was used to detect *Circovirus porcine2* (PCV2) in serum samples without the need for plate reading equipment. This process occurs by adapting the conventional ELISA test with gold nanoparticles (AuNPs) to promote a color change on the plate and quickly identify this difference with the naked eye, generating a dark purple-gray hue when the samples are positive and red when the samples are negative. The technique demonstrated high efficiency in detecting samples with a viral load ≥ 5 log_10_ copies/mL. Plasmonic ELISA offers user-friendly, cost-effective, and reliable characteristics, making it a valuable tool for PCV2 diagnosis and potentially adaptable for other pathogen detection applications.

## 1. Introduction

The presence of infectious diseases in swine has far-reaching consequences, affecting not only the livestock breeding industry and international trade but also posing risks to human public health [1]. Porcine circoviruses (PCVs) are recognized as one of the most widespread viruses worldwide. These viruses are classified under the family *Circoviridae*, genus *Circovirus*. They possess an icosahedral shape with a diameter of 17 nm and harbor a single-stranded circular DNA genome covalently closed. The PCV comprises four species: *Circovirus porcine1* (PCV1), *Circovirus porcine2* (PCV2), *Circovirus porcine3* (PCV3), and *Circovirus porcine4* (PCV4) [2].

Epidemiological research has consistently demonstrated that PCV2 is the primary causative agent in porcine circovirus-related diseases [3]. However, a literature review published by Denner and Mankertz (2017) showed that when PCV2 was transmitted through contaminated vaccines administered to children, no pathogenic effects were observed. This evidence suggests that there is no risk to human health [4].

PCV2 is transmitted through direct contact with infected animals and through the exchange of nasal, oral, urine, fecal fluid, oropharyngeal, colostrum, and semen. PCV2 can infect wild and domestic pigs, which may have varying health statuses [5]. The diagnosis of porcine circovirus disease (PCVD) is frequently performed by evaluating clinical signs [6] and identifying antigens or nucleic acids via PCR [7] or quantitative PCR (qPCR) [8], immunofluorescence [9], immunohistochemistry [10], in situ hybridization [11], immunoperoxidase [12], or conventional enzyme-linked immunosorbent assay (ELISA) [13]. The main limitation of all these techniques is that they require specific equipment to be operated by specialized individuals.

As an alternative, the development of colorimetric methodologies for the rapid detection of various types of samples without the need for specific instruments is underway [14,15]. Among the most commonly used materials in colorimetric tests are gold nanoparticles (AuNPs), which exhibit unique optical and electronic properties and are inexpensive; moreover, they are easy to synthesize and disperse in long-term solution and easy to store [16]. Usually, AuNPs change color through two processes: the reduction of HAuCl_4_ into atomic gold using enzymatic reagents and products (from colorless to red) or through interparticle binding, which leads to the aggregation of AuNPs (from red to blue/purple) [15,17,18]. Combining AuNPs with traditional techniques such as ELISA has been increasingly used to detect various analytes. For example, Ma et al. (2021) reported a multicolorimetric ELISA biosensor integrated with a paper/polymer hybrid microfluidic device for rapid visual detection of disease biomarkers at the point of care. The limit of detection of 9.1 ng/μL hepatitis C virus core antigen, a biomarker for hepatitis C, was achieved using a platform [19]. Weng et al. (2022) detected C-reactive protein (CRP), which is present in the blood during periods of inflammation, using plasmonic ELISA. The detection limit was determined to be 0.09 ng/mL with a spectrometer in the range from 0.09 to 25 ng/mL. The visual detection limit was 0.78 ng/mL, which allows differential diagnosis via the naked eye [20]. Dela Rica and Stevens (2012) used plasmonic ELISA to detect the HIV-1 capsid antigen p24, and prostate-specific antigen (PSA) was detected in whole serum at an ultralow concentration of 1 × 10^−18^ g/mL [21].

In conventional ELISA tests, enzymatic biocatalysis generates a colored compound that needs to be read with a specific reader to obtain reliable results. Therefore, the objective of this study was to identify PCV2 in serum samples using AuNPs in the ELISA test to promote a color change on the plate and quickly identify this difference with the naked eye, generating a dark purple-gray hue when the samples are positive and a red hue when the samples are negative.

## 2. Materials and Methods

### 2.1. Chemicals and Antibodies

Gold (III) chloride trihydrate (HAuCl_4_) 99%), 2-(N-morpholino) ethanesulfonic acid (4-morpholinoethanesulfonic acid; MES pH 6.0), hydrogen peroxide solution (H_2_O_2^−^_), Tween^®^20 (Saint Louis, MO, USA), carbonate–bicarbonate buffer (0.05 M, pH 9.6), and phosphate-buffered saline (PBS, pH 7.4) were purchased from Sigma-Aldrich (Saint Louis, MO, USA), and skim milk powder (SMP) was purchased from Elegê (Minas Gerais, Brazil).

Anti-pig IgG (whole molecule) * peroxidase (A5670) conjugate antibody was purchased from Sigma-Aldrich (Saint Louis, MO, USA).

Pig IgG anti-PCV2 (a detection antibody) was obtained from animals monitored for PCV via the Molecular Diagnostic Laboratory at São Paulo State University, Botucatu Campus, Brazil, and was obtained from a pool of sera positive for PCV2 IgG. These sera (*n* = 4) were tested individually by conventional ELISA and selected because they had a high ELISA index (a high titer of the IgG against PCV2).

Anti-PCV2 rabbit IgG (capture antibody) was produced by our research group for use in conventional ELISA for the detection of antibodies against PCV2. In this conventional ELISA, an antibody was produced by immunizing a female rabbit with purified PCV2 (50 µg/mL, *v*/*v*) by gradient ultracentrifugation in cesium chloride. Afterward, the serum obtained from the animals was purified using a HiTrap^®^ Protein G 5 mL column (GE Healthcare, Buckinghamshire, UK). The protein concentration was determined (8 mg/mL) using the bicinchoninic acid (BCA) method and bovine serum albumin (BSA) as a protein standard (protocol number 103/2010-CEUA-Ethics Committee Animal Use of FMVZ, São Paulo State University, Botucatu Campus) [13]. In all the experiments, water was sourced from a Millipore unit (Merck, Burlington, MA, USA).

### 2.2. Instruments

All washing steps during the experiment were performed in a BioTek™ EL × 50™ plate washer. A microplate StripWasher was used (BioTek^®^ Instruments, Winooski, VT, USA). Optical density (OD) was measured at 540 nm (Multiskan EX, Labsystems, Vienna, VA, USA). All the experiments were performed at 22 °C.

UV–Vis graphics were generated with a Biochrom Lira S11 Ultraviolet–Visible spectrophotometer (Biochrom Ltd., Cambridge, UK). Spectra were acquired using a glass cuvette with wavelengths ranging from 400 to 800 nm. Deionized water was used prior to each measurement to establish the reference spectrum.

The characterization of the AuNPs was performed using transmission electron microscopy (TEM) and energy dispersive X-ray (EDX) techniques on a JEM-2100 (JEOL Ltd., Peabody, MA, USA). ImageJ software was used to calculate the mean nanoparticle diameter (version 1.8.0_172). Dynamic light scattering (DLS) was performed using a Dyna PRO Titan (Wyatt Technology, Goleta, CA, USA) at a temperature of 30 °C with 100 acquisitions of 10 s each.

The swine serum samples were quantified using a qPCR instrument (7500 Fast Real-Time PCR System, Thermo Fisher Scientific; Foster City, CA, USA).

### 2.3. Swine Serum Samples Were Quantified by Quantitative Polymerase Chain Reaction (qPCR)

In total, 30 positive and 18 negative swine serum samples for PCV2 were tested by qPCR for quantification analysis. All samples used in this study were provided by the Molecular Diagnostic Laboratory at São Paulo State University, Botucatu Campus, Brazil, and originated from swine serum collection.

DNA extraction was performed using an Illustra Genomic Prep Mini Spin Kit (GE Healthcare, Buckinghamshire, UK) following the manufacturer’s instructions. qPCR was performed with primers described by Ladkjaer-Mikkelsen (2002) [22]. Reactions were prepared using 0.2 µM of each primer with GoTaq^®^ qPCR Master Mix kit (Promega, Madison, WI, USA). A standard curve plasmid was diluted from 10^8^ to 10^11^ DNA copies/mL in duplicate to determine the number of DNA copies. The results are presented as log_10_ DNA copies/mL to facilitate the visualization of the qPCR data (Table S1 in the supporting information).

### 2.4. Synthesis of Gold Nanoparticles

Based on the published works of De la Rica and Stevens (2012) [16] and Han et al. (2018) [15], HAuCl_4_ solutions were prepared and tested at concentrations of 0.1, 0.2, 0.4, and 1 mM and diluted in 1 mM 2-ethanesulfonic acid (MES) buffer at pH 6.0. The reducing agent used to form the AuNPs was H_2_O_2^−^_ (hydrogen peroxide) at a concentration of 240 µM, which was diluted in 1 mM MES buffer at pH 6.0.

To improve the results and ensure the formation of spherical, homogeneous, and dispersed AuNPs, different concentrations of H_2_O_2^−^_ (20, 40, 60, 80, 100, 120, 140, 160, 180, 200, 220, 240, 260, 280, and 300 µM) were added to the 1 mM HAuCl_4_ solution.

To confirm the synthesis, transmission electronic microscopy (TEM) images were analyzed with ImageJ software, and energy dispersive X-ray (EDX) and dynamic light scattering (DLS) analyses were performed to determine the final composition of the AuNPs.

### 2.5. Dilutions of the Conjugated Antibodies

To determine the optimal conjugate antibody dilution, initial tests were conducted on microtubes. For this purpose, 150 µL of the conjugate at a dilution of 1:250 was added to a centrifuge microtube, followed by the addition of 150 µL of H_2_O_2^−^_ (100 µM in MES buffer), and the mixture was incubated for 15 min at room temperature. Subsequently, 150 µL of gold solution (HAuCl_4_ in MES) was added to the microtube and incubated for 15, 30, 45, or 60 min.

Different dilutions of the conjugates at alternative time points were tested to determine the optimal parameters for ELISA experiments. The tested dilutions included 1:250, 1:500, 1:1000, 1:2000, 1:4000, and 1:8000 in MES buffer at 0, 15, 30, 45, and 60 min at room temperature.

To replicate the conditions, the dilution of the conjugate, which was previously performed in MES buffer, was tested in phosphate-buffered saline supplemented with 0.05% Tween 20 (PBST) at pH 7.4 at room temperature and 37 °C for different incubation times. To determine the optimal buffer and temperature, phosphate-buffered saline without Tween 20 (PBS) (pH 7.4) at room temperature and 37 °C and MES buffer (pH 6.0) at 37 °C were also tested. All analyses were conducted using a spectrophotometer.

### 2.6. Nanoparticle Growth in the Sandwich ELISA

In the literature, sandwich ELISAs were performed as described by Alhajj et al. (2023) in four main steps: coating, blocking, detection, and final reading [23]. For ELISAs using AuNPs, the protocol employed was based on the work of Cruz et al. (2016), who detected antibodies against PCV2 [13].

Polystyrene plates (Nunc-ImmunoPlateorp) with 96 wells were incubated with 50 µL of anti-PCV2 rabbit IgG (capture antibody—0.005 mg/mL) diluted in 0.05 M carbonate/bicarbonate buffer (pH 9.6) per well. The immobilization process was carried out at a temperature of 4 °C for 18 h.

The following day, the plate was washed five times with PBS and then blocked with 300 µL of solution per well to prevent nonspecific binding. A 10% (*w/v*) solution of skim milk powder (SMP) in 0.05 M carbonate/bicarbonate buffer was used for blocking. When the plates tend to have a positive charge, it is necessary to use an alkaline buffer (carbonate–bicarbonate buffer) so that the proteins become negatively charged and are attracted to the plastic on the plates. The plate was incubated in a humid chamber at 37 °C for 1 h. In the next step, the plate was washed five times with PBS, after which 50 µL was added to each well of serum samples obtained from pigs that were confirmed to be positive or negative for PCV2 via qPCR. The samples were diluted 1:200 in PBS containing 10% SMP and incubated at 37 °C for 1 h. Once again, the plate was washed five times with PBS and incubated at 37 °C for 1 h with 50 µL of pig IgG anti-PCV2 (detection antibody) diluted 1:500 in PBS containing 10% SMP per well. After incubation, the plate was washed five times with PBS.

**Figure 1:**
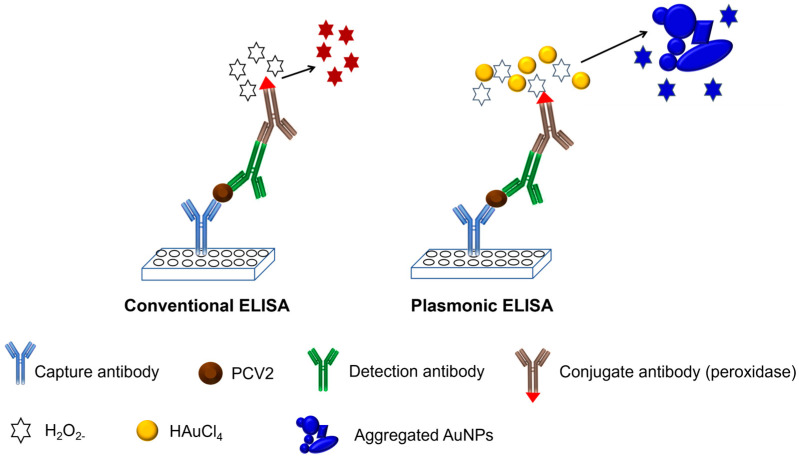
Schematic representation of the conventional ELISA and development of pELISA.

Subsequently, 50 µL of conjugate antibody conjugated with peroxidase was added to each well at a dilution of 1:250 in PBS. The plate was incubated at 37 °C for 1 h, followed by washing five times with PBS. Next, 50 µL of a 100 µM H_2_O_2^−^_ solution in 1 mM MES was added to each well as a substrate, after which the mixture was allowed to incubate for 15 min. Then, 50 µL of a 1 mM gold solution (1 mM MES) was added to each well, and the formation of nanoparticles was observed after 15 min. If the sample used contained the target analyte, the formation of a sandwich during ELISA would cause the substrate (H_2_O_2^−^_) to be rapidly consumed by the peroxidase enzyme, reducing the gold to AuNPs inefficiently. This results in nanoparticles with various shapes and a large number of aggregates, exhibiting blue/purple coloration. In contrast, if the desired sample did not contain the target analyte, the formation of the ELISA sandwich would not occur, and after the washing steps, the conjugated antibody would be removed. Consequently, the added H_2_O_2^−^_ remained free and reduced the subsequently added gold solution, generating spherical, proportionally sized, and nonaggregated AuNPs, resulting in red coloration. A schematic representation of the entire experiment is shown in Figure 1.

### 2.7. Interference Analyses

The efficiency of the color change was analyzed by testing serum samples from animals with varying viral loads, which were read at 540 nm to measure their optical density (OD). To validate the specificity of the proposed methodology, tests were conducted with potential interferents that could generate false-positive results. Along with PCV1, which is a nonpathogenic species commonly found in swine and similar to PCV2, viruses from the Adenoviridae and Parvoviridae families that present nonenveloped DNA were isolated from the testes [24,25,26].

## 3. Results and Discussion

**Figure 2:**
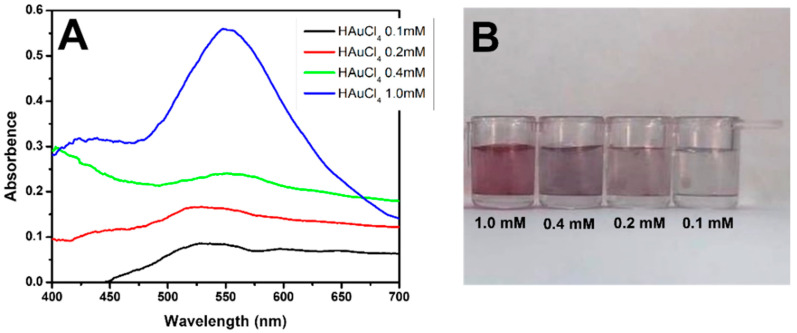
Formation of AuNPs. (**A**) UV–Vis spectra of HAuCl_4_ solution at concentrations of 0.1 mM (black line), 0.2 mM (red line), 0.4 mM (green line), and 1.0 mM (blue line). (**B**) Coloration of the solutions at different concentrations (1.0, 0.4, 0.2, and 0.1 mM).

Our detection system is based on the formation of gold nanoparticles. The synthesis of AuNPs using different concentrations of HAuCl4 in MES is shown in Figure 2A. The absorbances and wavelengths of the samples indicated that the nanoparticles were formed only at a concentration of 1 mM, which was also characteristic of the reddish color of AuNPs (Figure 2B).

**Figure 3:**
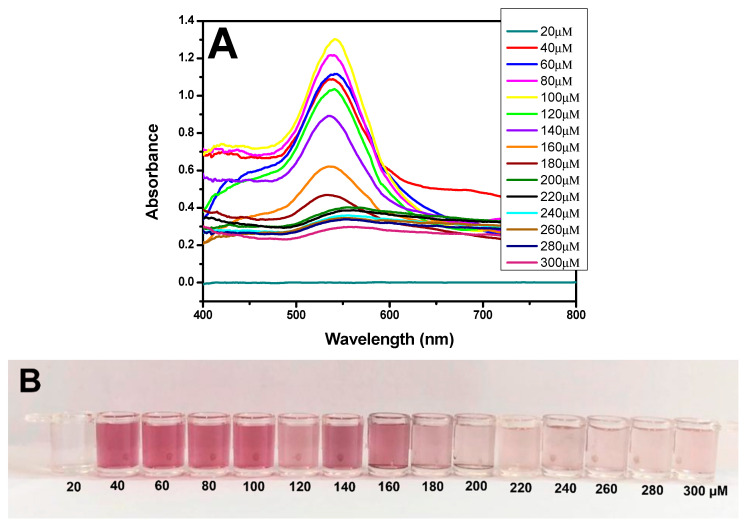

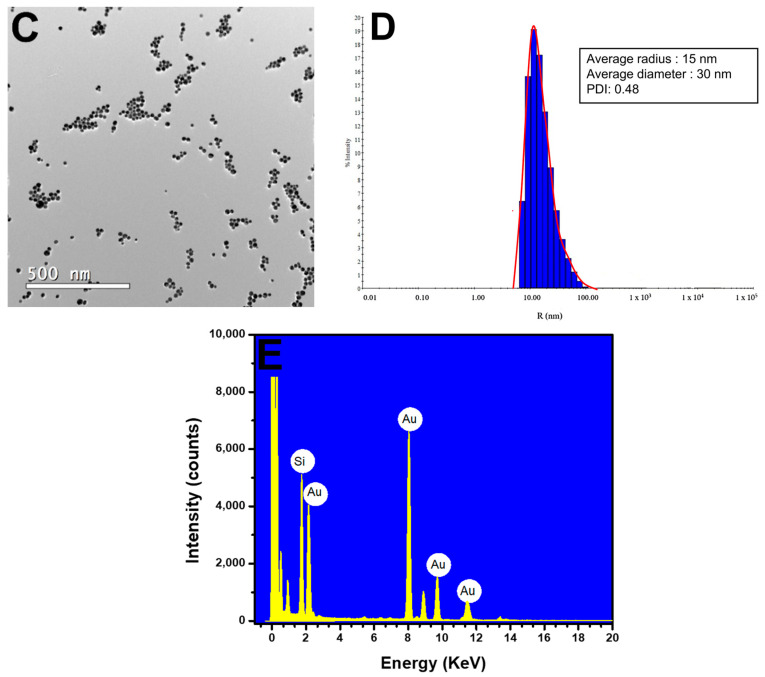
Characterization of AuNPs. (**A**) UV–Vis spectra of AuNPs at different concentrations of H_2_O_2^−^_ (20–300 µM). (**B**) Picture showing the color difference for synthesized AuNPs at various concentrations of H_2_O_2^−^_ (20–300 µM). (**C**) TEM image showing the AuNPs formed from 100 µM H_2_O_2^−^_ on a 500 nm scale. (**D**) DLS image showing the average diameter of the AuNPs at 30 nm. (**E**) EDX image shows the presence of gold (Au) in the samples.

The use of H_2_O_2^−^_ as a reducing agent for the formation of AuNPs was tested at different concentrations. A comparison of the results shown in Figure 3A revealed a greater peak at the yellow line, with an absorbance of 1.30 and a wavelength of 540 nm; these findings are characteristic of the formation of AuNPs, which impart a strong red color (Figure 3B). These data demonstrated that the synthesis of nanoparticles was correct. Therefore, the remaining experiments were conducted using 1 mM HAuCl_4_ (diluted in MES) reduced by a solution of 100 µM H_2_O_2^−^_ (diluted in MES).

TEM analyses revealed spherical AuNPs with an average diameter of approximately 28.3 nm (Figure 3C). The results were further confirmed by dynamic light scattering (DLS) analysis, which showed a mean diameter of 30 nm for the AuNPs, with a polydispersity index (PDI) of 0.48. The polydispersity index (PDI) indicates the heterogeneity of sizes in the sample, with a higher index indicating a more significant difference in sizes among the AuNPs (Figure 3D). Research published by Sharma et al. (2018) and Ghosh et al. (2019) concerning AuNPs showed PDI values similar to those found in this methodology [27,28]. The EDX results showed the presence of gold (Au) and silica (Si), which were attributed to the glassware used in the synthesis (Figure 3E).

**Figure 4:**
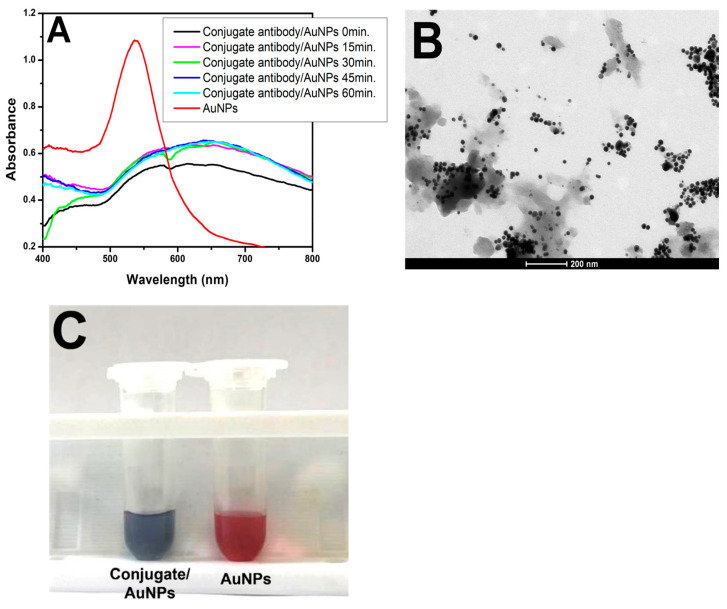
Conjugate with AuNPs. (**A**) UV–Vis spectra of the conjugate/H_2_O_2^−^_/gold solution at different times (black, pink, green, dark blue, and light blue lines) and the AuNP solution (H_2_O_2^−^_ and gold solution) for comparison (red line). (**B**) TEM image of the conjugate/H_2_O_2^−^_/gold solution on a 200 nm scale. (**C**) Picture of the conjugate/H_2_O_2^−^_/gold solution showing its purple/gray color and the red color of the AuNPs.

Subsequently, to initiate the pELISA test, the formation of AuNPs was tested in the presence of antibodies (Abs). A conjugate antibody against peroxidase was used (Figure 4A). The spectrophotometer readings at each tested incubation time revealed a gray coloration of the solution, indicating that the nanoparticles were aggregated and irregularly shaped. This occurred because the peroxidase enzyme in the conjugate rapidly consumed the H_2_O_2^−^_ added to the microtube. Consequently, when the gold solution was introduced, there was insufficient H_2_O_2^−^_ for AuNP formation. TEM images confirmed the irregular formation of nanoparticles (Figure 4B). To validate this observation, a second microtube was prepared containing only H_2_O_2^−^_ (100 µM in MES buffer) and gold solution (HAuCl_4_ in MES). In these microtubes, H_2_O_2^−^_ entirely reduced the amount of gold solution, resulting in spherical and dispersed AuNPs characterized by pink coloration (Figure 4C).

Following confirmation of the efficiency of AuNP formation in gold and H_2_O_2^−^_ solutions and the consumption of hydrogen peroxide by peroxidase, different dilutions of the conjugate at various times were examined to identify the optimal parameters for ELISA experiments. After evaluating the absorbance and wavelength of the samples shown in Figure S1A–F (Supplemental Materials) in conjunction with the colors in Figure S2 (Supplemental Materials), the optimal dilution of the tested conjugate was determined to be 1:250. The peaks at 15, 30, 45, and 60 min remained close together with minimal variation, and the resultant sample revealed a dark purple/gray color, characteristic of aggregated nanoparticles with irregular shapes. These results were consistent with those reported by Han et al. (2018), who used a biotinylated goat anti-rabbit IgG for roctopamine detection [10].

The dilution of the conjugate, which was previously performed in MES buffer, was tested in phosphate-buffered saline supplemented with 0.05% Tween 20 (PBST), and the results demonstrated that the use of PBST interfered with the consumption of hydrogen peroxide by the peroxidase enzyme. This interference led to the complete utilization of H_2_O_2^−^_ in reducing gold, which formed AuNPs. This effect was validated by the defined absorbance peaks (Figure S3A–F) and the colorations of the conjugate solutions, which exhibited the red hue characteristic of AuNPs at all dilutions (Figure S4—Supplemental Materials).

The use of PBST buffer at room temperature did not yield satisfactory results. MES buffer (pH 6.0) at room temperature previously showed promising results. MES at 37 °C yielded satisfactory results, as it did not interfere with the consumption of H_2_O_2^−^_ by the peroxidase enzyme of the conjugate, resulting in the formation of variously shaped and aggregated nanoparticles, as shown in Figure S5A. The same result was observed when using PBS at room temperature and at 37 °C (Figure S5B,C). However, when PBST was used at 37 °C, AuNPs formed without the consumption of peroxidase, as illustrated in Figure S5D, where well-defined absorbance peaks at approximately 540 nm can be observed. For comparison purposes, the measurements of AuNPs synthesized without the conjugate (pink lines) are also included in all the graphs. These results are visually distinguishable, with solutions using MES and PBS appearing grayish-purple, while the PBST solution exhibited a reddish hue, similar to the color of the formed AuNPs (Figure S6—Supplemental Materials).

Since the conventional ELISA test uses PBST buffer in its washing steps and for diluting the conjugate, after analyzing the previous results, dilutions of the conjugate in PBS buffer at room temperature and 37 °C were tested, along with H_2_O_2^−^_ and gold solution. Upon examining the results, we observed that both PBS at room temperature and 37 °C yielded similar results (Figures S7 and S8). Comparing the absorbance and wavelength values for PBS at room temperature with the photos of the solution colors, we found that the highest consumption of H_2_O_2^−^_ by peroxidase occurred at a dilution of 1:250 (Figure S9). The same trend was observed when comparing the consumption of peroxidase diluted in PBS at 37 °C, with darker shades occurring at lower dilutions (Figure S10). The plate test for the pELISA continued with dilutions of the conjugate in PBS at room temperature.

Having defined the optimal concentrations of the gold solution, H_2_O_2^−^_, and the dilution of the conjugate, the next step was to apply the solution to the ELISA system and observe the colorimetric changes. Samples with different virus concentrations were used, and the results could be interpreted with the naked eye (see Figure S11 for supporting information).

### Detection of PCV2 in the Serum by Plasmonic ELISA

Visual analysis in a traditional ELISA format has the potential to serve as a robust and cost-effective alternative to nucleic acid-based tests for PCV2 diagnosis.

The efficiency of the change in color of the cells was analyzed through tests on samples with viral loads ranging from 17.681 to 9.0 × 10^8^ copies/mL in positive samples; the results are presented in Table 1. The results are displayed as log_10_ values to facilitate more effective visualization of the qPCR data.

**Figure 5:**
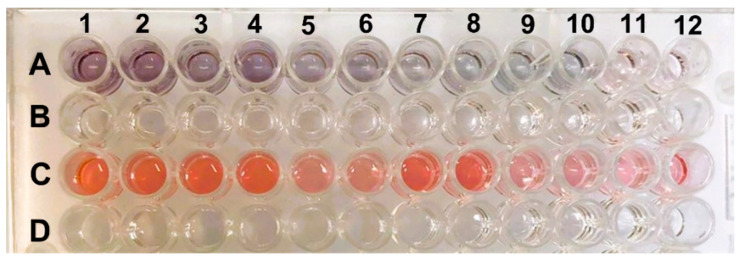
ELISA plate with positive and negative samples. Row (**A**) shows the positive samples with viral loads ranging from 17,681 to 9.0 × 10^8^ copies/mL, and row (**C**) shows the negative controls, which are samples without the presence of the virus. The lines (**B**,**D**) are empty spaces to facilitate the visualization of lines (**A**,**C**).

Figure 5 shows images of ELISA plates with different shades of purple and red. These colorations correspond to the duplicate viral loads of the samples. Samples with viral loads of 8 log_10_ copies/mL exhibited a darker purple color, while samples with 4 log_10_ copies/mL displayed a lighter color with a slight pink hue. When comparing the color obtained with the viral load, it was observed that higher virus concentrations in the sample produced darker purple coloration, and the hue became lighter as the viral load decreased. This trait can be visually detected more accurately up to 5 log_10_ copies/mL, where it showed a light purple hue (A9 and A10).

Row C in the ELISA plate shows the negative controls, which are samples without the presence of the virus. All the wells were red, indicating the correct formation of AuNPs (gold nanoparticles). The purple displayed by the positive samples can be easily differentiated from the red presented in the samples without antigen due to the biocatalytic action of peroxidase. The coloration is also directly proportional to the detected optical density. Darker samples (with a higher viral load) had lower OD values. The negative samples that correctly formed AuNPs exhibited red coloration and a lower OD than did the positive samples. This result is similar to what was found by Han et al. (2018) [15].

**Figure 6:**
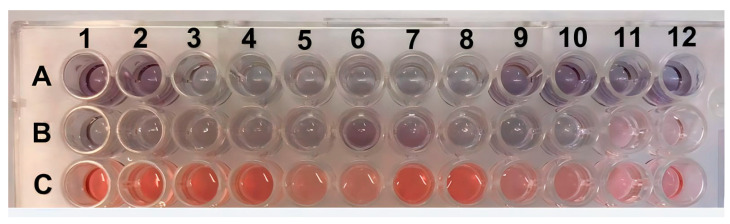
ELISA plate with positive samples in rows (**A**,**B**) (viral load ≥ 5 log_10_ copies/mL) and negative samples (≤4 log_10_ copies/mL viral load or without the presence of PCV2) in row (**C**). All samples were tested in duplicate and, they are indicated by the numbers.

**Figure 7:**
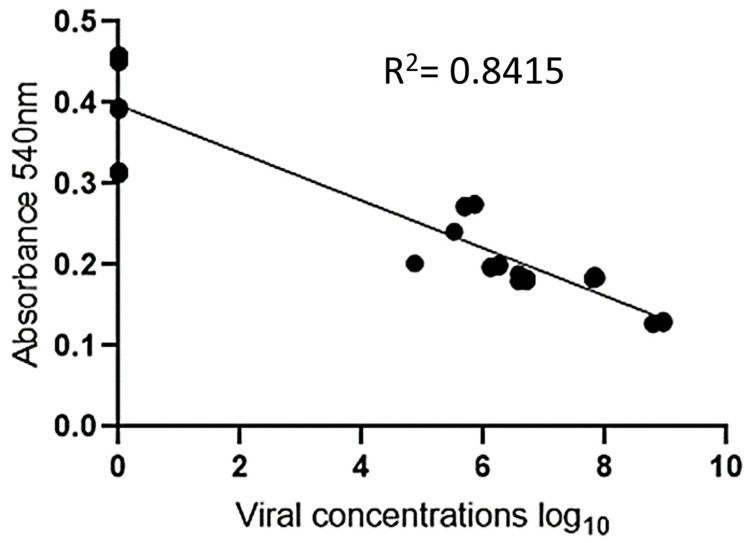
Graphical representation of linear regression for viral concentration (log_10_) on the X-axis vs. absorbance on the Y-axis.

The experiment was validated by testing 30 positive serum samples (ranging from 4 to 8 log_10_ copies/mL) and 18 negative serum samples. To facilitate visualization, twelve positive samples and six negative samples were analyzed in duplicate, and their absorbance measurements are displayed in Figure 6 and Figure 7. Photos of the other positive and negative samples are shown in Figures S12 and S13 (please refer to Supplementary Materials). The visual results on all plates showed that when the sample was positive with a viral load ≥ 5 log_10_ copies/mL, the H_2_O_2^−^_ solution was consumed by the peroxidase enzyme, resulting in asymmetric and variously sized purple nanoparticles. In contrast, the resulting color was red for samples with a 4 log_10_ copies/mL viral load or negative samples. Since there was no antibody conjugate in the wells, H_2_O_2^−^_ allowed for the efficient formation of AuNPs (Figure 6). These results indicate the absence of PCV2 or a low viral load in the tested samples. Figure 7 shows the correlation between the concentration and absorbance at 540 nm.

An analysis of the results revealed that when the sample was negative, it exhibited a red hue and higher absorbance than did the positive samples, which showed a purple tint. This result is similar to that found by Wang et al. (2017) when detecting cucumber green mottle mosaic virus using gold nanoparticles as colorimetric probes [29].

In comparison to traditional sandwich ELISAs, which typically use the chromogens 3,3′,5,5′-tetramethylbenzidine (TMB) and o-phenylenediamine dihydrochloride (OPD), which are highly toxic, the use of H_2_O_2^−^_ as a substrate does not pose health risks. The ELISA method using AuNPs is potentially suitable for qualitative analysis of the presence of PCV2 in unknown swine serum samples. All samples tested in this study were previously tested using qPCR.

A visual change in ELISA coloration with varying concentrations of AuNPs was also observed in the work of Wu et al. (2019), who developed a sandwich pELISA for *Cronobacter* detection in powdered infant formula samples, with a cutoff limit of 3 × 10^5^ cfu/mL [30].

Tests with potential interferents from the porcine adenovirus (serum samples) and canine parvovirus 2 (stool samples), both with 8 log_10_ copies/mL, showed that all of them exhibited a red color, indicating the formation of spherical and dispersed AuNPs in the absence of the target antigen. Because no sandwich structure was formed, the peroxidase enzyme did not consume H_2_O_2^−^_. In Figure S14 (Supplementary Materials), numbers 1 and 2 on the ELISA plate correspond to the porcine adenovirus samples, numbers 3 and 4 represent canine parvovirus 2 samples, and numbers 5 and 6 represent PCV1 samples. These samples were tested in duplicate wells.

A great advantage in this technique is its practicality and ease of detection since it does not require reading equipment to interpret the results, as also reported in the research by Maciel et al. (2020), who used indirect plasmonic ELISA for the detection of *Leishmania* spp. infection, which presented greater specificity than the official recommended method by the Ministry of Health in Brazil [31]. Although PCV2 causes a persistent infection in animals, its detection in blood samples is hindered by its short viremia. Therefore, tissue samples such as lymph nodes may be an alternative for a more prolonged detection of PCV2 using the pELISA method and will be tested in a subsequent stage.

## 4. Conclusions

The present study demonstrated the development of a new methodology for detecting PCV2. In this research, conventional ELISA was adapted with AuNPs to achieve a colorimetric reaction, the color change after the addition of 1 mM HAuCl_4_ solution diluted in 1 mM MES, which can show visually the presence of the virus. Positive serum samples exhibited a purple hue, while negative samples showed red coloration. However, the technique has limitations in accurately quantifying the concentration of the target molecule, especially for samples with similar shades, such as those with 6 log_10_ copies/mL and 7 log_10_ copies/mL. Therefore, the methodology proves to be a promising and effective tool for qualitatively analyzing the presence of the virus. Additionally, the technique can be adapted and employed to detect pathogens using the naked eye.

## Figures and Tables

**Table 1:** The number of PCV DNA copies/mL was quantified via quantitative PCR in the ELISA plate.

ELISA Plate Identification	Sample Identification	Quantification (Copies/mL)	Log_10_ (Copies/mL)
A1	SO8795	9.1 × 10^8^	8.96
A2	SO8795	9.1 × 10^8^	8.96
A3	SO8790	1.86 × 10^8^	8.27
A4	SO8790	1.86 × 10^8^	8.27
A5	SO8823	5.45 × 10^7^	7.74
A6	SO8823	5.45 × 10^7^	7.74
A7	SO10767	3.64 × 10^6^	6.56
A8	SO10767	3.64 × 10^6^	6.56
A9	SO12669	3.73 × 10^5^	5.57
A10	SO12669	3.73 × 10^5^	5.57
A11	SO16857	4.33 × 10^4^	4.64
A12	SO16857	4.33 × 10^4^	4.64
C1	SO12671	0	0
C2	SO12671	0	0
C3	SO12672	0	0
C4	SO12672	0	0
C5	SO12673	0	0
C6	SO12673	0	0
C7	SO12674	0	0
C8	SO12674	0	0
C9	SO12675	0	0
C10	SO12675	0	0
C11	SO12676	0	0
C12	SO12676	0	0

## Data Availability

The original contributions presented in the study are included in this article and Supplementary Material, further inquiries can be directed to the corresponding author.

## Supplementary Materials

The following supporting information can be downloaded at https://www.mdpi.com/article/10.3390/pathogens13020108/s1, Table S1: Number of porcine circovirus DNA copies per milliliter (mL) quantified by quantitative PCR (qPCR); Figure S1: Graphs showing the absorbance and wavelength at different dilutions of conjugates and AuNPs. (A) Conjugate at a 1:250 dilution. (B) Conjugate at a 1:500 dilution. (C) Conjugate at a 1:1000 dilution. (D) Conjugate at a 1:2000 dilution. (E) Conjugate at a 1:4000 dilution. (F) Conjugate at a 1:8000 dilution; Figure S2: Picture showing the differences in color among the samples with different dilutions of the conjugate in H_2_O_2^−^_ and the gold solution; finally, the AuNP solution was obtained using only the H_2_O_2^−^_ and gold solution; Figure S3: UV–Vis plot for different conjugate dilutions in PBST. (A) Conjugated at 1:250. (B) Conjugated at 1:500. (C) Conjugated at 1:1000. (D) Conjugated at 1:2000. (E) Conjugated at 1:4000. (F) Conjugated at 1:8000; Figure S4: Coloration of different dilutions of the conjugate in PBST; Figure S5: Comparison of the use of different buffers and temperatures in the 1:250 conjugate dilution. (A) MES at 37 °C. (B) PBS at room temperature. (C) PBS at 37 °C. (D) PBST at 37 °C; Figure S6: Conjugate 1:250 diluted in MES at 37 °C, PBS at room temperature, PBS at 37 °C, PBST at 37 °C, and AuNPs without the conjugate for comparison; Figure S7: Graphs demonstrating the wavelengths and absorbances of different dilutions of the conjugate in PBS buffer at room temperature at different incubation times. (A) Conjugate 1:250. (B) Conjugate 1:500. (C) Conjugate 1:1000. (D) Conjugate 1:2000. (E) Conjugate 1:4000. (F) Conjugate 1:8000; Figure S8: Graphs demonstrating the wavelengths and absorbances of different dilutions of the conjugates in PBS at 37 °C after different incubation times. (A) Conjugate 1:250. (B) Conjugate 1:500. (C) Conjugate 1:1000. (D) Conjugate 1:2000. (E) Conjugate 1:4000. (F) Conjugate 1:8000; Figure S9: Colorimetric image of the different dilutions of the conjugate in PBS buffer at room temperature; Figure S10: Colorimetric image of the different dilutions of the conjugate in PBS buffer at 37 °C; Figure S11: ELISA plate with positive serum samples in row A, negative serum samples in row B, and controls in rows C–F; Figure S12: Samples of serum from different animals that tested positive for PCV2; Figure S13: Samples of serum from different animals that tested negative for PCV2; Figure S14: Analysis of potential interferents. 1 and 2 adenovirus, 3 and 4 parvovirus, and 5 and 6 PCV1 samples.
