# Supplementary material for: Development of a Gold Nanoparticle-Based ELISA for Detection of PCV2
Source: Pathogens. 2024 Jan 26;13(2):108. doi: 10.3390/pathogens13020108 (PMC10893201; doi:10.3390/pathogens13020108)
Supplement: Supplementary file 1 [file pathogens-13-00108-s001.zip › pathogens-2752609-supplementary.pdf]

Modification of the Conventional ELISA Test for Visual detection of PCV2 Virus Using Gold Nanoparticles

Caroline Rodrigues Basso [1,*], Taís Fukuta Cruz [1, 2], Larissa Baldo Vieira[1] , Valber Albuquerque Pedrosa [2], Fabio Sossai Possebon[1,] and João Pessoa Araújo Júnior [1,2].

[1] Biotechnology Institute, São Paulo State University- Botucatu, SP 18607-440, Brazil; caroline.basso@unesp.br (C.R.B); tfcruz@yahoo.com.br (T.F.C.); larissa.baldo@unesp.br (L.B.V); fabio.possebon@unesp.br (F.S.P) and joao.pessoa@unesp.br (J.P.A.J.).

[2] Chemical and Biological Sciences Department, Bioscience Institute, São Paulo State University- Botucatu, SP 18618-000, Brazil; valber.pedrosa@unesp.br (V.A.P).

Table S1: Number of Porcine circovirus DNA copies per mililiter (mL) quantified by quantitative PCR (qPCR).

Sample Identification	Quantification (copies/mL)	Log_{10} (copies/mL)
SO16857	4.33×10^4	4,64
SO16942	7.41×10^4	4,87
SO10754	2.61×10^5	5,42
SO16954	3.33×10^5	5,52
SO12669	3.73×10^5	5,57
SO16948	4.95×10^5	5,69
SO16946	7.00×10^5	5,85
SO16945	1.31×10^6	6,12
SO16949	1.82×10^6	6,26
SO8791	2.65×10^6	6,42
SO10767	3.64×10^6	6,56
SO16956	3.80×10^6	6,58
SO16961	5.09×10^6	6,71
SO16943	1.36×10^7	6,13
SO8798	4.85×10^7	6,69
SO8823	5.45×10^7	6,74
SO5G1	6.31×10^7	7,80
S16955	6.33×10^7	7,80
SO8810	6.74×10^7	7,83
SO16959	6.83×10^7	7,83
SO11974	8.89×10^7	7,95
SO8799	1.29×10^8	8,11
SO8800	1.51×10^8	8,18
SO8790	1.86×10^8	8,27
SO8788	2.41×10^8	8,38
SO16944	2.57×10^8	8,41
SO8802	3.77×10^8	8,58
SO16947	6.23×10^8	8,79
SO8787	8.24×10^8	8,92
SO8795	9.10×10^8	8,96

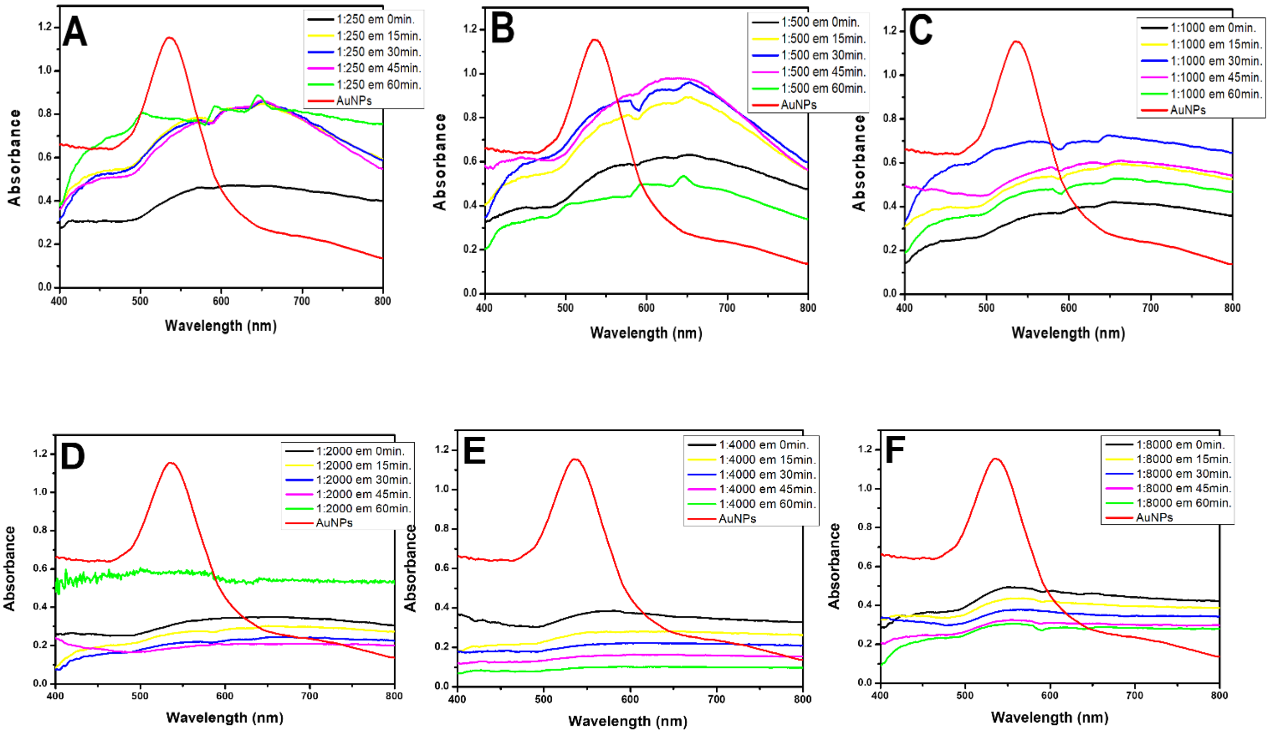

Figure S1: Graphs showing absorbance and wavelength at different dilutions of conjugate and AuNPs. **A-** Conjugate in 1:250 dilution. **B-** Conjugate in 1:500 dilution. **C-** Conjugate in 1:1,000 dilution. **D-** Conjugate in 1:2,000 dilution. **E-** Conjugate in 1:4,000 dilution. **F-** Conjugate in 1:8,000 dilution.

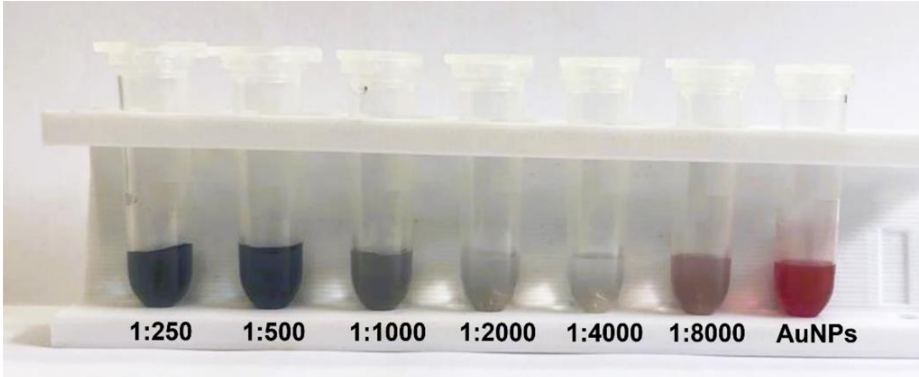

Figure S2. Picture showing the differences in colors among the samples with different dilutions of the conjugate in H_2O_2- and the gold solution, and finally, the AuNP solution obtained using only the H_2O_2- and gold solution.

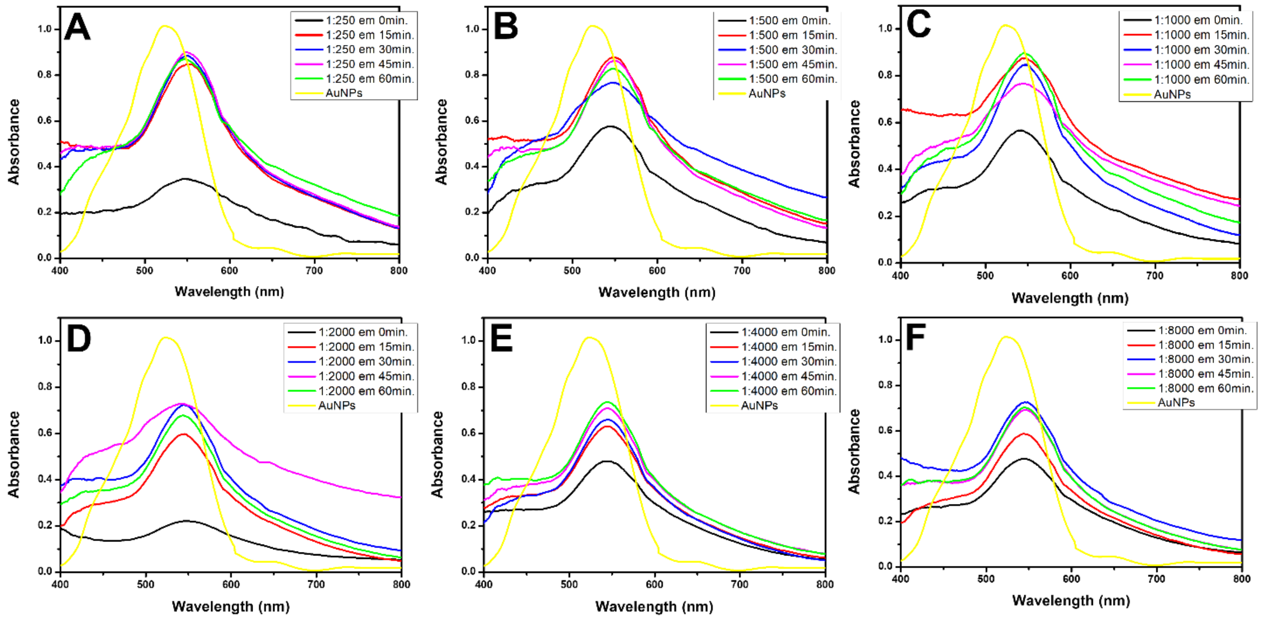

Figure S3: UV-Vis plot for different conjugate dilutions in PBST. **A-** Conjugated at 1:250. **B-** Conjugated at 1:500. **C-** Conjugated in 1:1,000. **D**- Conjugated in 1:2000. **E-** Conjugated in 1:4,000. **F-** Conjugated in 1:8,000.

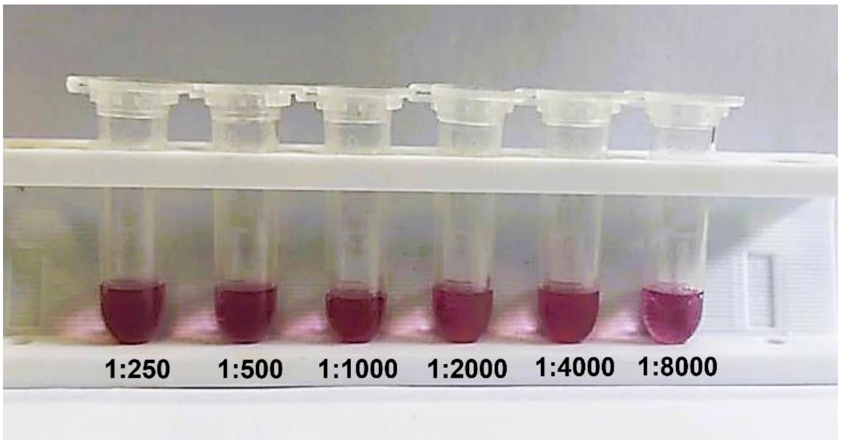

Figure S4. Coloration of different dilutions of the conjugate in PBST.

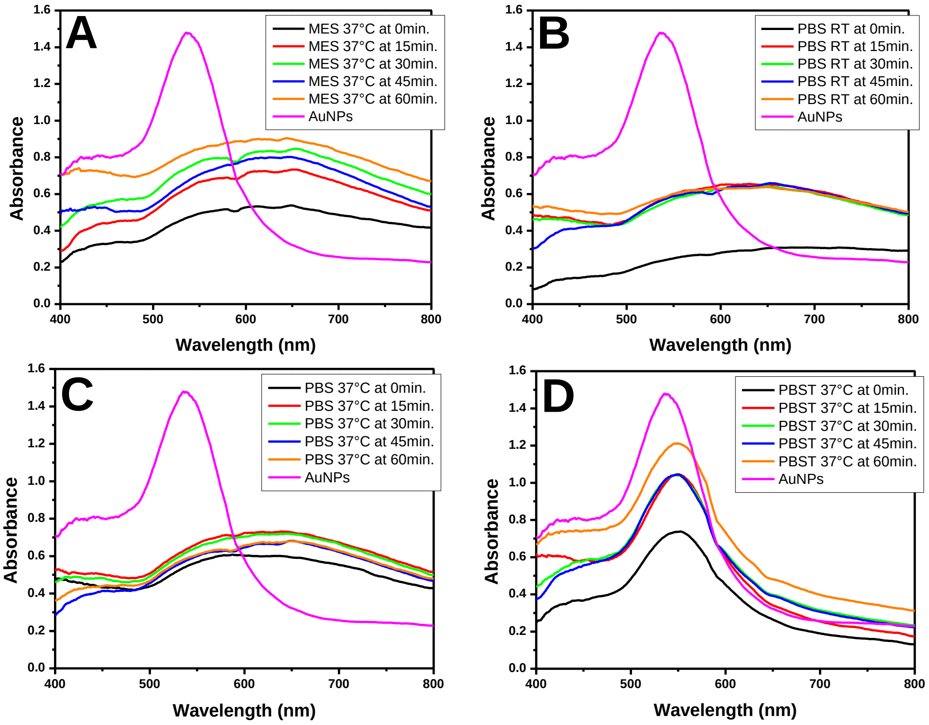

Figure S5: Comparison of the use of different buffers and temperatures in the 1:250 conjugate dilution. **A-** MES at 37°C. **B-** PBS at room temperature. **C-** PBS at 37°C. **D-** PBST at 37°C.

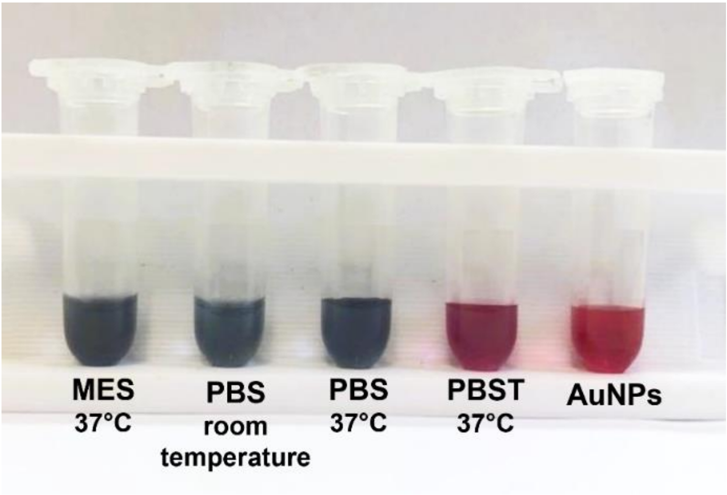

Figure S6. Conjugate 1:250 diluted in MES at 37°C, PBS at room temperature, PBS at 37°C, PBST at 37°C, and AuNPs without the conjugate for comparison, respectively.

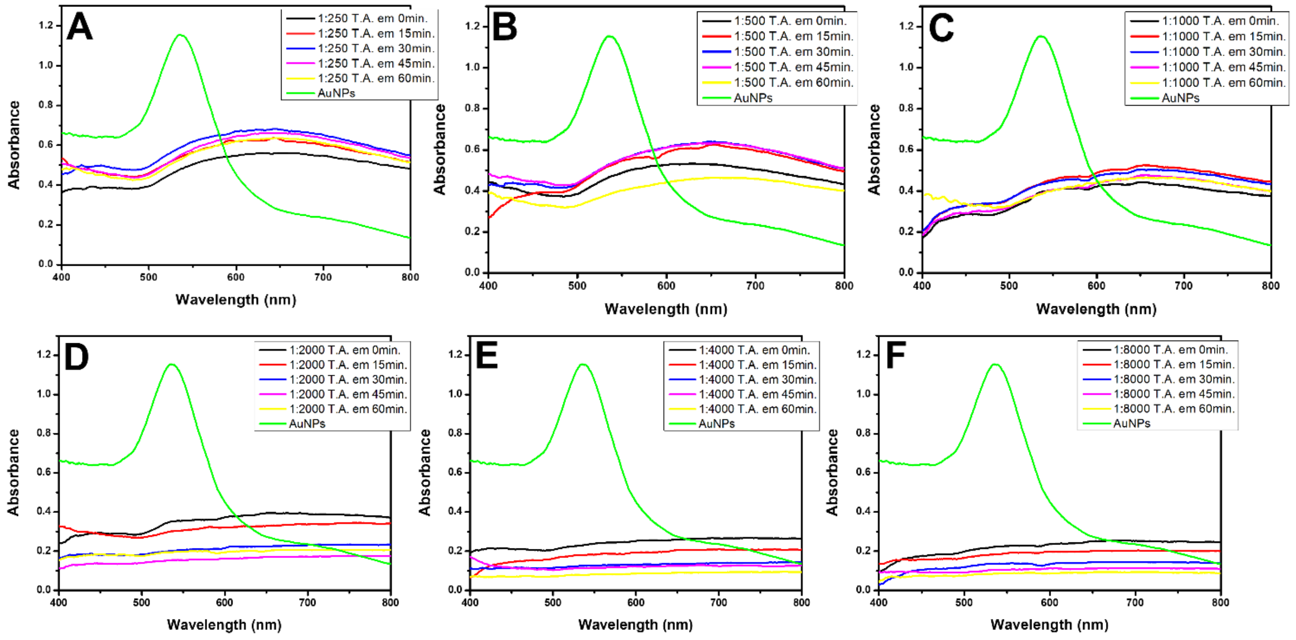

Figure S7: Graphs demonstrating the values of wavelengths and absorbances of different dilutions of the conjugate in PBS buffer at room temperature at different incubation times. **A-** Conjugate 1:250. **B-** Conjugate 1:500. **C-** Conjugate 1:1,000. **D-** Conjugate 1:2,000. **E-** Conjugate 1:4,000. **F-** Conjugate 1:8,000.

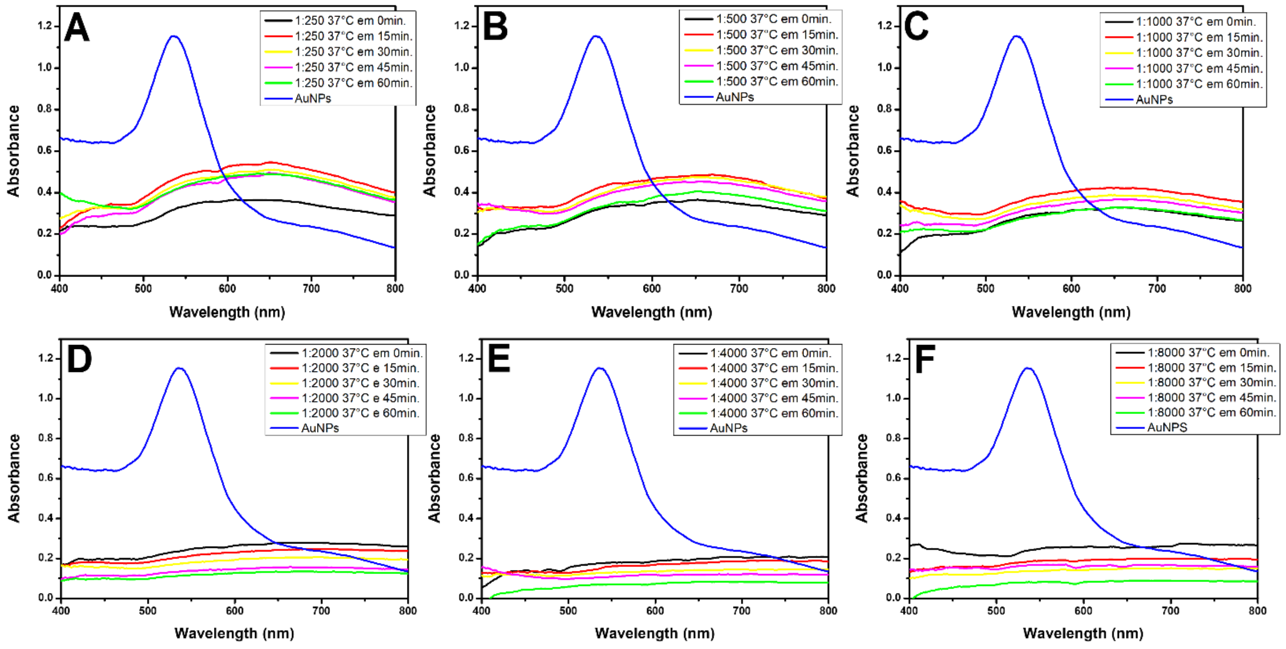

Figure S8: Graphs demonstrating the values of wavelengths and absorbances of different dilutions of the conjugate in PBS buffer at 37°C at different incubation times. **A-** Conjugate 1:250. **B-** Conjugate 1:500. **C-** Conjugate 1:1,000. **D-** Conjugate 1:2,000. **E-** Conjugate 1:4,000. **F-** Conjugate 1:8,000.

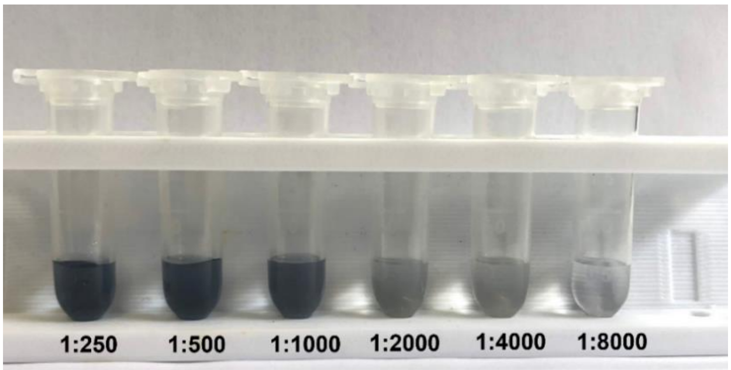

Figure S9. Colorimetric image of the different dilutions of the conjugate in PBS buffer at room temperature.

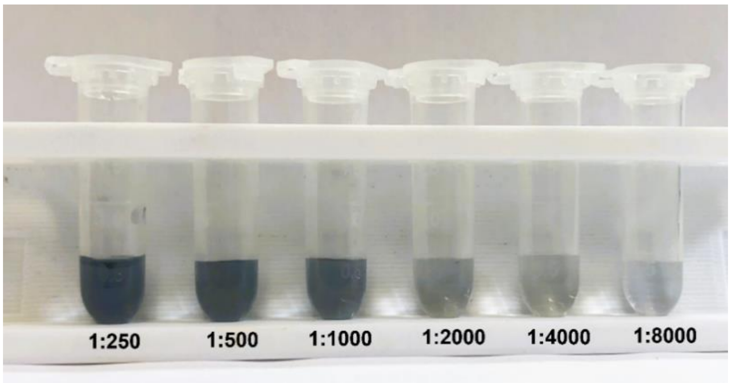

Figure S10. Colorimetric image of the different dilutions of the conjugate in PBS buffer at 37°C.

In the ELISA plate, wells A1 and A2 correspond to a duplicate positive sample for PCV with a 8 log_{10} copies/mL, showing a dark purple color. Wells A3 and A4 are from a duplicate positive sample with a 6 log_{10} copies/mL concentration, presenting a light purple/blue tone. In both samples, the color change to a purple shade indicates the consumption of H_2O_{2-} by peroxidase, confirming the qPCR result. Wells B1-B2 and B3-B4 are two distinct duplicate negative samples. The red color observed indicates the correct formation of spherical, dispersed AuNPs of similar sizes, confirming the negative result of the qPCR. The wells in rows C, D, E, and F correspond to the experimental controls and represent:

C1: capture antibody, C2: positive serum 8 log_{10} copies/mL, C3: negative serum only, C4: detection antibody, C5: conjugate, C6: H_2O_2, C7: gold solution, C8: capture

antibody/ positive serum 8 log_{10} copies/mL, C9: capture antibody/ negative serum, C10: capture antibody/ detection antibody, C11: capture antibody /conjugate, C12: capture antibody/ H_2O_{2-}.

D1: capture antibody/ gold, D2: positive serum 8 log_{10} copies/mL/ detection antibody, D3: positive serum 8 log_{10} copies/mL/ conjugate, D4: positive serum 8 log_{10} copies/mL/H_2O_{2-}, D5: positive serum 8 log_{10} copies/mL/gold, D6: negative serum/ detection antibody, D7: negative serum/ conjugate, D8: negative serum/ H_2O_{2-}, D9: negative serum/ gold, D10: detection antibody/ conjugate, D11: detection antibody/ H_2O_2, D12: detection antibody/ gold.

E1: conjugate/ H_2O_{2-}, E2: conjugate/ gold, E3: $H_{2}O_{2-}$/ gold, E4: capture antibody/ positive serum 8 log_{10} copies/mL/ detection antibody, E5: capture antibody/ negative serum/ detection antibody, E6: capture antibody/ positive serum 8 log_{10} copies/mL/ conjugate, E7: capture antibody/ positive serum 8 log_{10} copies/mL/ H_2O_{2-}, E8: capture antibody/ positive serum 8 log_{10} copies/mL/ gold, E9: capture antibody/ negative serum/ conjugate, E10: capture antibody/ negative serum/ H_2O_{2-}, E11: capture antibody/ negative serum/ gold, E12: gold/ detection antibody/ H_2O_{2-}.

F1: positive serum 8 log_{10} copies/mL/ detection antibody/ H_2O_{2-}, F2: positive serum 8 log_{10} copies/mL/ detection antibody/ gold, F3: negative serum/ detection antibody/ conjugate, F4: negative serum/ detection antibody/ H_2O_{2-}, F5: negative serum/ detection antibody/ gold, F6: detection antibody/ conjugate/ H_2O_{2-}, F7: detection antibody/ H_2O_{2-} / gold, F8: conjugate/ H_2O_{2-} / gold.

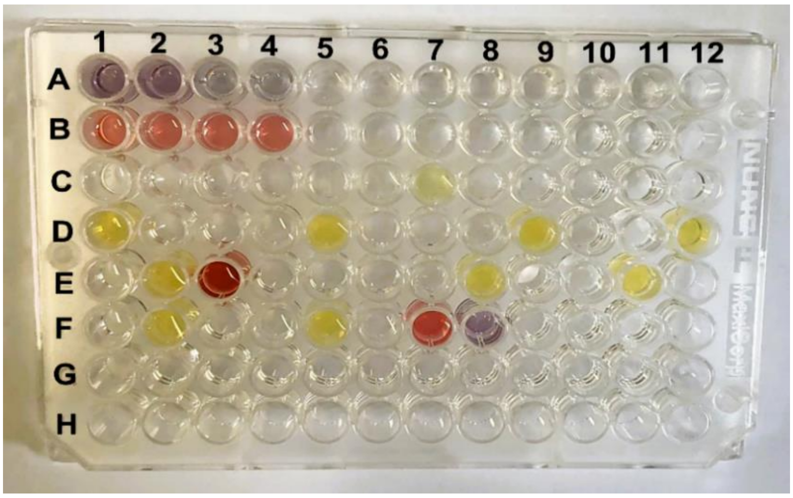

Figure S11: ELISA plate with positives serum samples in row A, negative serum samples in row B, and controls in rows C-F.

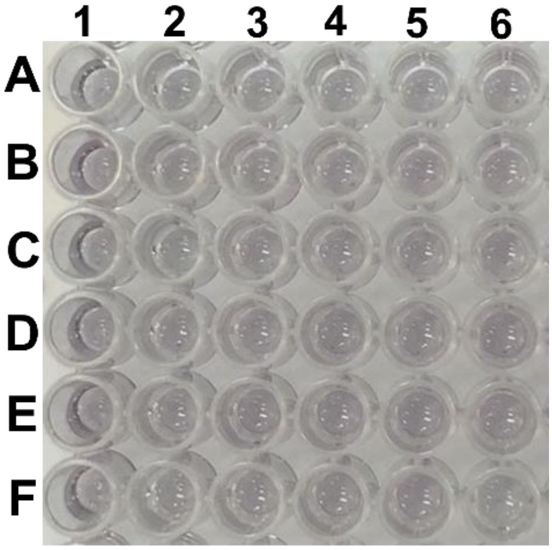

Figure S12: Samples of serum from different animals that tested positive for PCV2.

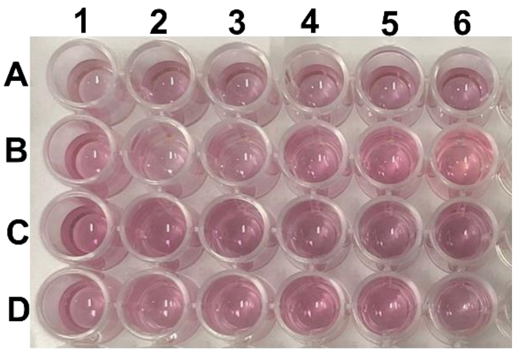

Figure S13: Samples of serum from different animals that tested negative for PCV2.

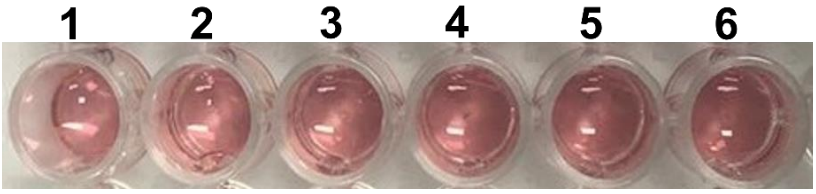

Figure S14. Analysis of potential interferents. 1 and 2 adenovirus, 3 and 4 parvovirus and, 5 and 6 PCV1 samples.
